# The Efficacy of Extracorporeal Shockwave Therapy Compared with Compression Therapy in Healing Venous Leg Ulcers

**DOI:** 10.3390/jcm13072117

**Published:** 2024-04-05

**Authors:** Paweł T. Dolibog, Patrycja Dolibog, Beata Bergler-Czop, Sławomir Grzegorczyn, Daria Chmielewska

**Affiliations:** 1 Department of Biophysics, Faculty of Medical Sciences in Zabrze, Medical University of Silesia, 19 H. Jordan Str., 41-808 Zabrze, Poland; grzegorczyn@sum.edu.pl; 2 Department of Medical Biophysics, Faculty of Medical Sciences in Katowice, Medical University of Silesia, 18 Medyków Str., 40-752 Katowice, Poland; pdolibog@sum.edu.pl; 3 Department of Dermatology, Medical University of Silesia in Katowice, 20-24 Francuska Str., 40-027 Katowice, Poland; bbergler-czop@sum.edu.pl; 4 Electromyography and Pelvic Floor Muscles Laboratory, Institute of Physiotherapy and Health Sciences, Department of Physical Medicine, The Jerzy Kukuczka Academy of Physical Education, 72a Mikołowska Str., 40-065 Katowice, Poland; d.chmielewska@awf.katowice.pl

**Keywords:** venous leg ulcers, venous ulcer treatment, physical treatment, elongation index

## Abstract

**Background**: Innovative methods of physical therapy delivered via modern medical devices have significantly extended the possibility of applying conservative treatments in healing venous leg ulcers. The primary objective of this study was to compare the therapeutic efficacy of selected mechanical physical therapies (intermittent pneumatic compression vs. radial extracorporeal shockwave vs. focal extracorporeal shockwave) vs. standard care in the treatment of venous leg ulcers over a 4-week period. **Materials**: This study included 69 patients, comprising 45 females (65%) and 24 males (35%), with a mean age of 67.1 ± 8.6 years (range: from 52.0 to 80.0 years). **Methods**: The patients were allocated into four groups: the IPC group was treated with intermittent pneumatic compression therapy, the R-ESWT group was treated with radial extracorporeal shockwave therapy, the F-ESWT group was treated with focal extracorporeal shockwave therapy, and the SC group was treated with standard care. **Results:** After one month of therapy, the median percentage decrease in wound total surface area after treatment was as follows: in the IPC group, there was a 52.9% decrease (range: 3.3–100%); in the R-ESWT group, there was a 31.6% decrease (range: 2.4–95.8%); in the F-ESWT group, there was an 18.0% decrease (range: 1.9–76.1%); and in the SC group, there was a 16.0% decrease (range: 1.5–45.8%). **Conclusions:** All the studied therapies caused a statistically significant reduction in the surface area of venous leg ulcers. The best results were observed with the intermittent pneumatic compression, while the radial and focal extracorporeal shockwave therapies appeared less effective. The standard care alone turned out to be the least effective. Our results did not show statistically significant changes in the values of RBC deformability at the investigated shear rates.

## 1. Introduction

Venous leg ulcers are a challenge for modern medicine. They affect as much as 1% of the adult population in developed countries, often affecting more women than men, and their incidence increases with age [1]. Most ulcerations last for more than half a year, and recurrences occur in over two-thirds of patients [2]. Patients’ quality of life with an ulceration is low as it often causes social exclusion and absenteeism from work, and the costs of patient care are constantly increasing [3].

It has been known for a long time that the direct cause of venous ulcers is chronic venous insufficiency; however, despite many studies by scientists and clinicians, effective and durable treatment methods are still being sought.

Under physiological conditions, 10–15% of the blood in circulation flows through the superficial vein system, while as much as 85–90% flows through the deep veins. The pressure due to open valves and relaxed muscles in both superficial and deep veins is high and is about 80–90 mmHg. The work contraction of the muscles causes blood to move from the superficial to the deep veins, causing a decrease in the pressure in the superficial veins (to about 10 mmHg) and an increase in the deep veins (200–300 mmHg). In the disease process, when the valves are leaky, about 25–30% of the blood backs up in the deep veins and flows into the superficial ones. This causes the transfer of high pressure to the superficial veins, thus resulting in their widening and a tortuous course for smaller venous vessels. This causes the deepening of valve failure and thus the stagnation of blood in the deep vein system [4,5].

Chronic venous insufficiency (CVI) is characterized by a permanent disorder of blood outflow through the veins of the lower limbs, varicose veins and edema, and the occurrence of pathological changes within the skin and subcutaneous tissue; consequently, venous ulceration occurs.

The aim of effective healing of leg ulcers is to lower the venous pressure and maintain it in the vessels of the lower limbs. The selection of physical methods, such as compression, sonotherapy, or shockwave therapy, is due to these methods being able to affect the abnormal rheological conditions in the blood vessels of the lower limbs through physical and mechanical interactions (factors) [6].

Compression therapy is the gold standard of care for venous ulcers and chronic venous insufficiency. The healing outcomes in clinical studies have demonstrated the efficient effects of compression therapy on leg ulcers [7,8,9]. Currently, there are many studies using graduated compression of the limbs, such as elastic bandages, Unna boots, multi-layer elastic compression bandages, short-stretch bandages, compression stockings, and intermittent compression therapy, but the therapeutic outcomes are not always clearly defined. Bandages and stockings primarily affect the amount of pressure at the ankle level (ranging from 30 to 40 mmHg) after they are worn for 8 to 12 h. Moreover, compression stockings are the most basic product used to prevent the recurrence of VLUs [9].

Active compression therapy devices are made of a cuff covering the foot, lower leg, and thigh. The cuff consists of four to twelve chambers filled sequentially with air. The pressure exerted at the ankle level is adjustable in the range of 15 to 120 mmHg; the air chambers are filled (30–95 s) and deflated (10–95 s); as a sequentially, as a wave, or as linked therapy cycles, which can be controlled. The treatment duration is 10–99 min [10,11].

The use of extracorporeal shockwave therapy (ESWT) for the treatment of chronic wounds is growing, with the highest usage found in Central European countries and the USA. The shockwave is characterized by a very high peak pressure (even 100 MPa) that rapidly increases (<10 ns) and then falls below the ambient pressure within 12 ms. The shockwaves are defined by their energy flux density, number and frequency of impulses, and pressure. In current clinical practice, the most popularly used modes of shockwave generation are piezoelectric and electrohydraulic generation. Shockwave propagation applicators can be divided into focused (F-ESWT—focused extracorporeal shockwave therapy) and radial categories (R-ESWT—radial extracorporeal shockwave therapy). Both focused and radial shockwave units have been utilized in the treatment of wounds, using typical energy levels of 0.037 to 0.3 mJ/mm^2^ and a frequency range of 1–8 Hz. F-ESWT’s depth of penetration is dependent on the use of a coupling cap and can reach 12 cm, while R-ESWT penetrates to a depth of approximately 3 cm [12].

According to the document from the European Wound Management Association (EWMA), extracorporeal shockwave therapy (ESWT), photobiomodulation (PMB), and nanotechnologies (NTs) were recognized as advanced therapies [13].

Blood and its properties are crucial for transporting nutritional products to tissues, thereby ensuring the proper functioning of the body. Proper blood supply is crucial in the treatment of CVI-related ailments [14]. Rheology deals with the study of blood properties under shear stress and serves as a diagnostic method in many diseases. Additionally, CVI may affect rheological indices related to obstructed blood outflow from the lower limbs and pathological changes in microcirculation [15]. Blood viscosity depends on hematocrit, plasma viscosity, red blood cell aggregation, and deformability. Red blood cells’ (RBCs) deformability can influence the properties of red blood cell flow independently of their effect on blood viscosity. Reduced RBC deformability may lead to increased resistance in the microcirculation and the entrapment of RBCs in various organs [16].

Reviews of results from clinical trials on the impact of compression and shockwave therapy on the healing of leg ulcers typically confirm their positive effectiveness. However, these reviews often lack direct comparison between the two therapies. Our decision to compare the effectiveness of these therapies stems mainly from the absence of such a comparison in the available literature, as well as concerns regarding the sometimes low methodological quality or incompleteness of the reports.

The primary objective of the study was to compare the therapeutic efficacy of selected mechanical physical therapies (intermittent pneumatic compression vs. radial extracorporeal shockwave vs. focal extracorporeal shockwave) vs. standard care in the treatment of venous leg ulcers over a 4-week period. The other objectives were to assess the elongation index (EI) of red blood cells (RBCs) in patients with VLUs.

## 2. Materials and Methods

### 2.1. Study Design

**Figure 1:**
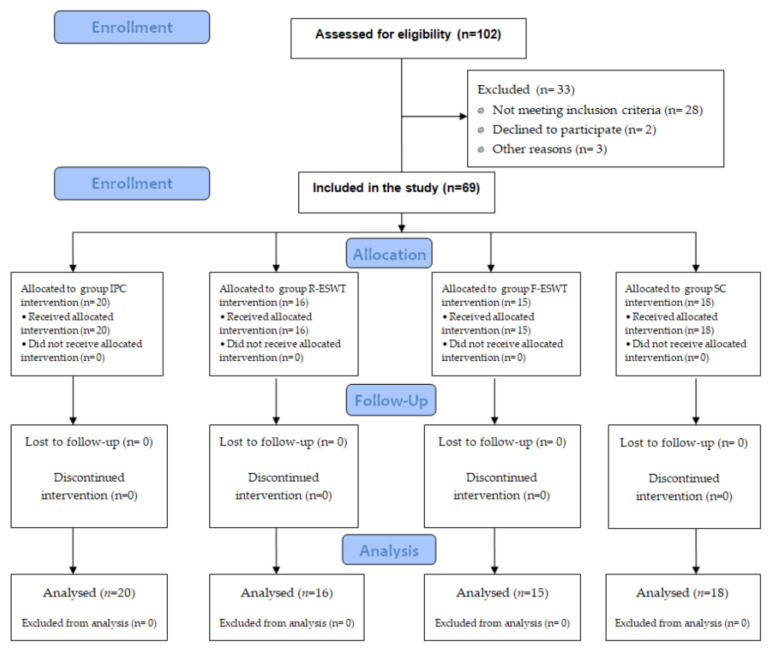
The CONSORT 2010 flow diagram of patients in the study.

A single-center randomized study was conducted at a center of dermatology (Department of Dermatology at the Medical University of Silesia) from December 2015 to December 2020. The project, methodology, and study were approved by the local Bioethical Committee of the Medical University Silesia (protocol number KNW/0022/KB1/25/II/15). All participants gave written informed consent before entering the study. The study was performed according to the requirements of the Consolidated Standards of Reporting Trials (CONSORT) statement (Figure 1). The patients with venous leg ulcer were divided into four groups, as follows:

(1) The IPC group (*n* = 20), in which patients were treated with intermittent pneumatic compression therapy; (2) the R-ESWT group (*n* = 16), in which patients were treated with radial extracorporeal shockwave therapy; (3) the F-ESWT group (*n* = 15), in which patients were treated with focal extracorporeal shockwave therapy; (4) the SC group (*n* = 18), in which patients were treated with standard care only.

### 2.2. Patients

We included patients with VLU diagnosed by a dermatologist and a vascular surgeon in order to confirm the disease. Each patient had an ultrasound examination of the arteries and veins of their lower limbs before the procedures were started. Additionally, the ankle brachial pressure index (ABPI) was established, which for all patients was higher than 0.8. Patients with VLUs persisting for more than one month in one lower limb were eligible for the study. The initial examination also assessed other clinical characteristics of the ulceration, including its warmth, redness with a diameter greater than 2 cm, swelling, presence of pus, unpleasant smell, and pain.

The exclusion criteria for the patients were as follows: surgical treatment of ulcers, atherosclerosis, diabetes, cancers, rheumatoid arthritis, ventricular arrhythmia, cardiac pacemakers, infections of the skin, pregnancy, implants from foreign bodies in the potential field of application, and age under 18 or over 80 years old.

In all groups, patients received standard care (SC): gauze dressing saturated in 0.9% sodium chloride and elastic bandages that were changed daily.

**Figure 2:**
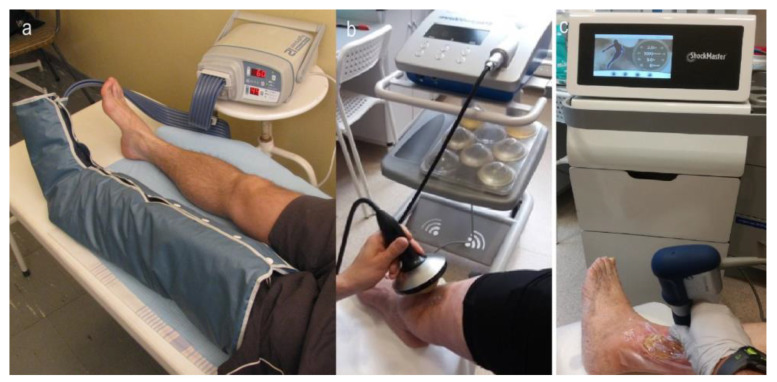
The procedure of therapy with the use of (**a**) intermittent pneumatic compression, (**b**) focused extracorporeal shockwave therapy, and (**c**) radial extracorporeal shockwave therapy.

The patients were assigned to study groups according to the order in which they reported to the dermatology clinic. The attending physician had no influence on the order of patient recruitment and the selection of the treatment method. Initially, patients were recruited for treatment using intermittent pneumatic compression therapy. Upon completion of this phase, patients were recruited to the radial extracorporeal shockwave therapy group. Subsequently, the focus shifted to focal extracorporeal shockwave therapy, targeting patients from the next group. After completing treatment using the physical methods, patients were recruited to the last group—standard care. Each patient could only be recruited to one group. The study was conducted according to the principles in the Declaration of Helsinki. Figure 2 shows photos of patients being treated with active compression therapy (a), focused shockwave therapy (b), and radial shockwave therapy (c).

### 2.3. Therapeutic Procedures

All patients in all groups were treated by dermatologists (treatment coordination), physiotherapists (compression therapy and shockwave therapy), and nurses (standard care, wound management). Prior to commencing each series of treatments, a technician inspected every therapeutic device for efficiency and reliability, ensuring optimal treatment parameters.

Patients from the IPC group received intermittent pneumatic compression therapy. The compression was administered using a Flowtron Hydroven System device (Huntleigh Healthcare, Cardiff, UK), with the cuff covering the leg from the foot to the thigh. All patients were subjected to a pressure of 60 mmHg at the ankle, which decreased to 40 mmHg at the thigh level. Treatments were conducted seven days per week (from Monday to Sunday) for a duration of four weeks, and a single treatment lasted 60 min.

Patients in the R-ESWT group received radial extracorporeal shockwave therapy. All treatments were administered using a ShockMaster 500 generator (Gymna Uniphy, Bilzen, Belgium) with a classic applicator of 15 mm. The treatment protocol consisted of radial waves with a pressure of 0.2 MPa, frequency of 5 Hz, impulse amount of 100 impulses/cm^2^, and surface energy density of 0.17 mJ/mm^2^. The R-ESWT was provided six times at intervals of 5 days over 4 weeks.

Patients in the F-ESWT group received focal extracorporeal shockwave therapy. All treatments were administered using a Piezowave generator (Elvation Medical, Kieselbronn, Germany) with an F10G4 applicator. The treatment protocol consisted of focused waves with a peak pressure of 35.6 MPa, frequency of 5 Hz, impulse amount of 100 impulses/cm^2^, and surface energy density of 0.173 mJ/mm^2^. The F-ESWT was provided six times at intervals of 5 days over 4 weeks.

The treatment in the R-ESWT and F-ESWT groups involved applying sterile gel to the wound for the transmission of mechanic waves, followed by the application of a sterile self-adhesive surgical foil (elastoFILM, Zarys, Poland). The gel was reapplied on top of the foil.

Patients in the SC group received only standard care for a period of 4 weeks.

### 2.4. Assessment

The wound healing rates were assessed on the basis of using high-resolution digital photographs with markers and non-contact digital planimetry. The non-contact digital planimetry was characterized by a high accuracy of measurements (the measurement error was less than 0.4%). AutoCad software v. 2013 was used to assess the total ulcer area (in cm^2^) and perimeter (in cm) of the wound [17].

The primary endpoint of the study outcome was wound relative change in the ulcer surface area and perimeter after 4 weeks of treatment. The following formulas were used to determine them: RCA (%) = 100 × (AW0 − AW4)/AW0, where RCA is the relative change in the ulcer surface area, AW0 is the baseline wound area, and AW4 is the wound area at week 4. Four measurement time points were assumed: immediately before (W0, baseline) and 1 (W1), 2 (W2), 3 (W3), and 4 (W4) weeks after the start of treatment.

The secondary endpoint of the study was the weekly wound healing rate (WHR) [18,19]. Using the linear formula WHR = ΔA/$\bar{P}$, the wound healing rate was calculated regardless of wound size (where ΔA represents the change in wound area between successive weekly measurements (cm^2^) and $\bar{P}$ represents the mean ulcer perimeter between successive weekly measurements (cm)).

RBC deformability was measured using a Lorrca laser optical device (RR Mechatronics, Zwaag, The Netherlands) at a stable temperature of 37 ± 0.2 °C. The measurement of RBC deformability involved mixing 12.5 μL of blood with 2.5 mL of polyvinylpyrrolidone buffer solution. Deformability was measured using erythrocyte elongation indices in the shear stress range from 0.3 to 30 Pa. The elongation index (EI) was calculated using the following equation: EI = (A − B)/(A + B), where A and B represent the major and minor radii of the axis of the ellipse, respectively. The EI allows the evaluation of erythrocyte elasticity and is calculated based on the change in erythrocyte shape under shear stress.

Blood samples for the study were collected before and after treatment. A detailed description of the preparation of blood samples and the determination of the EI was described in a previous publication [20].

### 2.5. Sample Size

Sample size analysis was performed using Statistica v 13.3 (TIBCO). We estimated the sample size necessary to demonstrate statistical significance between the study groups at *p* = 0.05 and a test power of 0.8, which was 60 patients.

### 2.6. Statistical Analysis

All statistical analyses were performed using STATISTICA software (v 13.3, TIBCO Software Inc., Palo Alto, CA, USA, 2017) and PQStat software (PQStat Software, v.1.8.4, Poznań, Poland). To avoid bias, all samples were anonymized and numbered. The normality of the distribution of the data was tested by means of the Shapiro–Wilk test. To compare variables in groups of patients, a chi-squared test of independence (the highest level of reliability), the ANOVA Kruskal–Wallis test, and the Kruskal–Wallis post-hoc test were used. To compare the measured values (WHR index) obtained before and at 1, 2, 3, and 4 weeks after therapy, the non-parametric Friedmann ANOVA test and post-hoc Dunn–Bonferroni test were used. A comparison of outcomes before (W0) and after 4 weeks (W4) of intervention in each group was performed using the non-parametric Wilcoxon test. The effect size (Cohen’s d) was also calculated, with d = 0.20 indicating a small effect, d = 0.50 indicating a medium effect, and d = 0.80 indicating a large effect. Statistical significance was assumed at a *p*-value of < 0.05.

## 3. Results

The final study included 69 patients whose characteristics were assessed before randomization. Among them, there were 45 females and 24 males with a mean age of 67.1 ± 8.6 years (range: 52.0–80.0 years) and BMI of 29.1 ± 5.5 kg/m^2^ (range: 20.3–42.9 kg/m^2^). Inefficiency of superficial veins was diagnosed in 55% of patients and that of superficial and deep veins was diagnosed in 45% of patients. Table 1 presents the demographic characterization of the study groups after randomization. The homogeneity of the groups was tested for the number of patients, sex, smoking, obesity, duration of ulceration, and age.

All patients were monitored for 28 days (4 weeks), during which the mean value of the ulcer surface area changed statistically significantly in all groups (main effect: *p* < 0.05), indicating the effectiveness of treatment across all groups. Complete healing of wounds was observed in two patients in the IPC group. Planimetric measurements were performed before treatment (W0) and at 1 (W1), 2 (W2), 3 (W3), and 4 (W4) weeks after the initiation of treatment.

**Figure 3:**
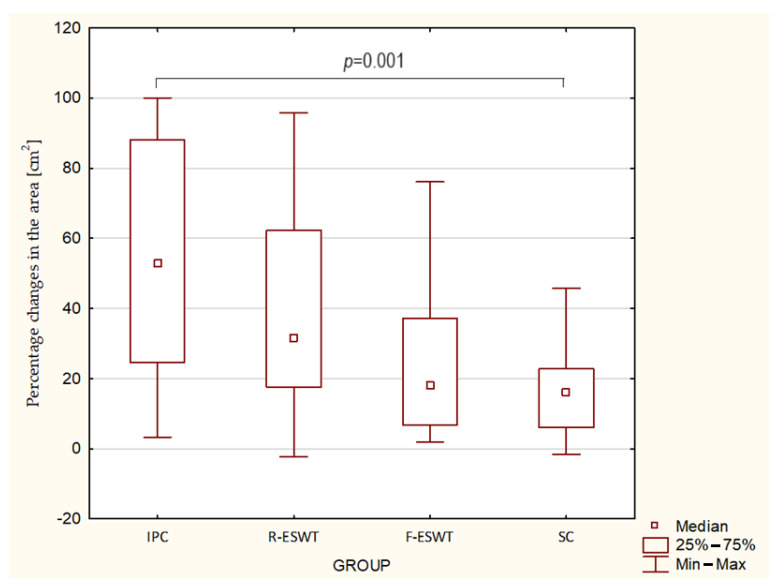
Comparison of mean percentage changes in the area (cm^2^) of ulceration in the study groups.

The relative percentage change in ulcer surface area for patients in the IPC group had a median of 52.9% (quartile range: 24.6–88.2%); for the R-ESWT group, it was 31.6% (quartile range: 17.6–62.3%); for the F-ESWT group, it was 18.0% (quartile range: 6.8–37.2%); and for the SC group, it was 16.0% (quartile range: 6.0–22.7%). Intergroup comparisons showed particularly favorable statistically significant clinical changes in patients treated with active compression therapy compared to patients receiving standard care (Table 2, Figure 3). The effect size for the IPC vs. F-ESWT, IPC vs. SC, and R-EWST vs. SC group comparisons was large; for the IPC vs. R-ESWT, R-ESWT vs. F-ESWT, and F-ESWT vs. SC group comparisons, there was a medium effect.

**Figure 4:**
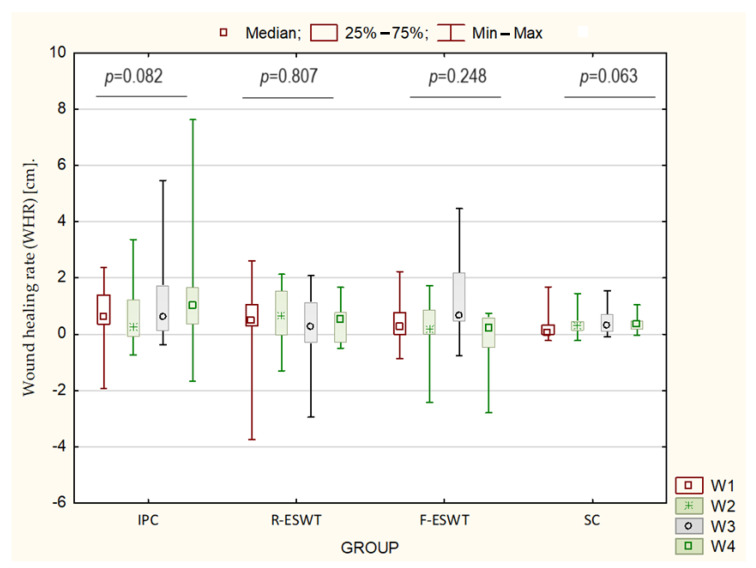
Comparison between groups in terms of wound healing rate (WHR) [cm].

The analysis of changes in the WHR index throughout each week of treatment showed that there were statistically significant differences in the healing rates between individual weeks of treatment. In the R-ESWT, F-ESWT, and SC groups, the healing rate was significantly lower in the 1st and 4th weeks of treatment compared to the IPC group. In the SC group, there were no differences in the value of WHR coefficient throughout the observation period. The analysis of changes in the percentage of WHR index and wound total surface area confirmed that intermittent pneumatic compression was the most efficient treatment, and the radial and focused shockwave therapies also appeared to be much more effective compared to standard care (Table 3, Figure 4).

**Figure 5:**
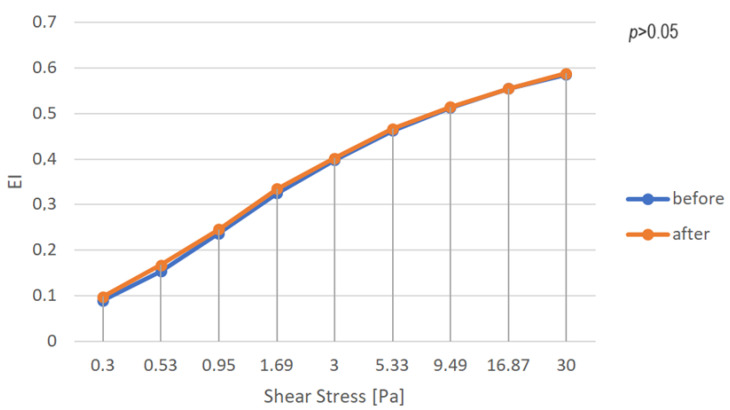
Dependence of the elongation index on the shear stress for blood samples immediately before and after therapy.

Our results indicate that there were no statistically significant changes in the values of RBC deformability at each of the investigated shear rates. Specifically, the EI value did not differ significantly between the beginning of therapy and its end at each of the wall pressures tested (*p* > 0.05) (Figure 5).

## 4. Discussion

Treatment of chronic wounds cannot be regarded through a binary system (wound healed) because the treatment process is very complex and has a multidimensional basis. Lots of studies nowadays are searching for very advanced conservative treatments for wound healing, such as physical therapy, pharmacological treatment, or dressing in hydrogels. The main goal of physical therapy is restoring the optimal skin function—that is, the healed wound structure.

This 4-week study demonstrated a significant improvement in the treatment of VLUs with IPC compared to SC. To the best of our knowledge, this study is the first to compare the healing rates of selected mechanical physical factors (compression therapy and shockwave treatments) in the treatment of VLUs. According to the findings of this study, the best results for percentage change in wound surface area were achieved in the group treated with IPC (52.9%), followed by radial (31.6%) and focal (18%) extracorporeal shockwave therapies, and the standard care was the least effective (16%). Under the influence of external pressure, the diameter of the veins decreases, the volume of venous blood in the limb decreases, blood flow is accelerated, and the quality of the skin and subcutaneous blood flow improves [21].

In addition to assessing the percentage change in the wound surface area, our study evaluated the WHR through weekly assessments. This approach was used due to the fact that the healing rate of wounds of different sizes is not the same [18,19,22]. The mean WHR coefficient was 9.6 mm in the IPC group, 6.1 mm in the R-ESWT group, 4.3 mm in the F-ESWT group, and 3.1 mm in the SC group per week.

There was a strong tendency for the WHR values to be higher in the IPC group as compared to the R-ESWT, F-ESWT, and SC groups. However, these differences were not statistically significant. In contrast, there were no differences in the WHR levels among the R-ESWT, F-ESWT, and SC groups. Naik et al. [23] demonstrated that an IPC device designed to be applied in the thigh region of the affected limb (patients with lower limb ulceration of both venous and mixed origin) resulted in wounds progressing towards healing in 95.24% (20/21) of the patients. The percentage reduction in the wound area from recruitment to 8-week follow-up was a mean of 43.75%, with the mean wound area having decreased from 19.02 cm^2^ to 13.15 cm^2^ (*p* < 0.000). The authors concluded that the thigh-applied IPC device can be recommended for use in routine clinical practice, because most patients felt that it was comfortable and easy to apply and remove.

The above results are consistent with a study conducted by Marston et al. [24] which compared the effects of a pneumatic compression device (ACT—active compression therapy group) and a multilayered bandage (MLB group) on wound healing. The study assessed the percentage changes in wound area. The results showed that ACT treatment had a greater effect on wound healing than did MLB (83.8% vs. 70.5%, respectively). The authors also concluded that this mode of therapy appears to have promise for improving the cost-effectiveness of treatment for chronic VLUs.

Alvarez et al. [25] compared the effects of IPC plus standard compression bandages versus standard compression bandages alone for the treatment of chronic VLUs. They found that the speed of healing (in mm/d) was more than two times greater (*p* = 0.41) in the group receiving both standard compression bandages and IPC therapy (1.7 ± 0.5 mm/d) compared to only standard compression bandages (0.8 ± 0.2 mm/d). The authors confirmed the effectiveness of IPC and suggested that IPC is a valuable adjunct to compression therapy in the management of large chronic VLUs.

Another systematic review of IPC devices suggested improved wound healing in cases of venous insufficiency when compared with no compression. However, the review emphasized the need for further well-designed studies to substantiate these positive results [26].

There are limited studies in the available literature confirming the effectiveness of ESWT in the treatment of VLUs. This is also confirmed by the latest Cochrane review, which notes that no randomized controlled trials (RCTs) have assessed the effectiveness of extracorporeal shockwave therapy in the healing and management of venous leg ulceration [27].

In both extracorporeal shockwave therapy (ESWT) study groups, therapy lasted for 4 weeks, with six treatments administered at one week intervals. Taheri et al., treated patients for 4 weeks with four treatments. Similarly, Aschermann et al., performed ESWT four times, once every 3–4 weeks [28]. Furthermore, Stieger et al., observed that after just five ESWT sessions, progressive wound granulation and epithelial renewal from the edges could be observed [29].

This study found that treatment with ESWT was generally well tolerated. This observation is consistent with findings from other researchers [29,30]. This suggests the safety and acceptability of these treatments for patients. Aschermann et al., emphasized in their research that, similar to our study, ESWT was well tolerated by patients, without the need for local anesthesia [28].

In our study, we used ESWT in both groups with a frequency of 5 Hz and 100 impulses for each cm^2^ of wound and used a surface energy density of 0.17 mJ/mm^2^ for R-ESWT and 0.173 mJ/mm^2^ for F-ESWT. These parameters were comparable to the studies of Taheri et al. (total energy, 3.5 mJ; frequency, 5 Hz; 100 pulses per cm^2^ of wound area), Aschermann et al. (100 impulses per cm^2^ + 200 impulses), and Stieger et al. (surface energy density, 0.25 mJ/mm^2^; frequency, 4 Hz; 2000 impulses). After four weeks of therapy, our study demonstrated a median reduction in wound area of 32.1% for radial and 18% for focused shockwave therapy. A similar result of reducing the wound surface area was obtained by Taheri et al., with radial waves after 4 weeks of use, with a reduction of 31.2%. Additionally, in the study group, the patients’ pain value was decreased more and the feeling of satisfaction was increased more compared to the control group. Swiss researchers evaluated the effectiveness of ESWT for chronic leg ulcers. The results showed that among the treated venous ulcers, 21 showed complete healing (49%), 7 showed significant improvement (16%), 12 improved (28%), and 3 did not improve (7%). In conclusion, the authors emphasized that EWST may advance the treatment of chronic venous leg ulcers [28,29,30].

The median wound healing rate showed positive trends across all treatments. The combination of mechanical factors with SC resulted in an increase in wound healing rates during this study period. The mean wound closure rate in our 4-week study was approximately 9.6 mm wound size reduction/week for IPC, 6.1 mm wound size reduction/week for R-ESWT, 4.1 mm wound reduction per week for F-ESWT, and 3.1 mm wound reduction per week for SC. The WHR is rarely reported in studies, so this parameter is difficult to compare, and it is an independent parameter for the duration of treatment.

In our study, we did not observe statistical differences in the EI before and after the treatments. This lack of significant difference is likely attributed to the observation period and the fact that not all wounds had healed by the end of the study. Nevertheless, this result is intriguing and warrants further investigation in blood rheology. Moreover, we did not find any studies in the available literature concerning RBC deformation in venous leg ulcers. Chwała et al., compared EI results for patients with CVI and healthy patients, which were statistically higher in the range of 1.13 to 8.23 Pa for shear stress in CVI patients compared to healthy people. This difference was explained by compensation for higher vascular resistance in people with CVI [31].

We could conclude that among patients with VLUs, the value of the elongation index does not change significantly after treatment. However, further studies are necessary to confirm our preliminary results and gain a better understanding of the impact of treatment on erythrocyte deformability in this patient population.

The cited studies demonstrate the effectiveness of compression therapy and shockwaves in promoting the healing of VLUs compared to standard care alone, which aligns with the results obtained in our study. However, our study goes further by comparing the effectiveness of these methods with each other, which is particularly important in the context of treatment planning. Supplementing standard treatment with additional mechanical methods is particularly important because they directly impact the rheological conditions in the venous vessels of the lower limbs, thus determining the effectiveness of treatment. It should be noted that in our study, we followed the treatment process in detail using the wound rate. The current study found that the selected mechanical physical factors did not affect the EI values in VLU patients, perhaps because this treatment period was too short to change the EI significantly. Further research is necessary to explain this question.

### Limitations

The present study has several limitations, which in our opinion require comments and discussion. Firstly, the study is limited by the small number of patients in the groups, and larger sample sizes might somewhat alter the results. In order to be independent of the size of the wound, we used the relative percentage change in ulcer surface area and wound healing rate (WHR) index for analysis. Another limitation of the study is the short observation time, but it was our intention to assess the physical methods in the short term to choose the most effective method of treatment for patients. Lastly, because of the way patients were assigned to a treatment group, there could be some bias to the results.

## 5. Conclusions

The use of mechanical physical factors (shockwave and active compression therapy) to aid the healing of venous leg ulcers significantly improved their healing. The most favorable outcomes were observed following intermittent pneumatic compression, while radial and focal extracorporeal shockwave therapies appeared less effective. Standard care alone turned out to be the least effective approach. It is necessary to conduct studies on a larger population.

## Figures and Tables

**Table 1:** Baseline characteristics of the patients in comparison groups. Abbreviations: 25Q—25th quartile; Me—median; 75Q—75th quartile; *—ANOVA Kruskal–Wallis test; **—chi-squared test.

Variable/Group	IPC	R-ESWT	F-ESWT	SC	*p*
Sample size	20	16	15	18	
Age [years] (%)					
<60	7 (35)	1 (7)	1 (7)	8 (44)	0.189 **
60–69	5 (25)	7 (43)	8 (53)	2 (12)	
70–79	8 (40)	8 (50)	6 (40)	8 (44)	
BMI [kg/m^2^]					
Me (25Q–75Q)	29.7 (26.7–36.0)	28.0 (25.9–30.5)	27.4 (24.2–29.6)	25.5 (22.9–31.2)	0.07 *
Sex, n (%)					
Female	14 (70)	11 (6)	5 (33)	15 (83)	0.162 **
Male	6 (30)	5 (31)	10 (67)	3 (17)	
Smokers n (%)	6 (30)	3 (19)	5 (33)	2 (11)	0.289 **
Localization of VLU					
Lateral ankle	9 (45)	7 (44)	2 (13)	9 (50)	
Medial ankle	11 (55)	9 (56)	8 (53)	7 (39)	0.058 **
Frontal surface	0	0	4 (27)	2 (11)	
Posterior surface	0	0	1 (7)	0	
Duration of VLU [months]					
Me (25Q–75Q)	8 (6–27)	10 (6–19)	12 (6–16)	18.5 (7–36)	0.337 *

**Table 2:** Comparison of the average values of the surface area of treated VLUs between the study groups before and after treatment. Abbreviations: RCA—relative percentage change in ulcer surface area; 25Q—25th quartile; Me—median; 75Q—75th quartile; *—ANOVA Kruskal–Wallis test; **—Wilcoxon test.

Variable	IPC Group	R-ESWT Group	F-ESWT Group	SC Group	p *
Initial wound size area [cm^2^]					0.652
Me (25Q–75Q)	5.4 (3.3–15.9)	6.1 (2.5–12.3)	7.4 (4.6–16.2)	10.5 (5.8–12.0)	
Final wound size area [cm^2^]					
Me (25Q–75Q)	1.3 (0.3–10.9)	3.5 (1.4–7.9)	7.1 (1.9–10.3)	9.7 (4.4–10.8)	0.087
RCA [%]					
Me (25Q–75Q)	52.9 (24.6–88.2)	31.6 (17.6–62.3)	18.0 (6.8–37.2)	16.0 (6.0–22.7)	0.001
*p* **	<0.001	<0.001	<0.001	<0.001	

**Table 3:** Comparison of the change in the average WHR index between the study groups after the first (W1), second (W2), third (W3), and fourth (W4) weeks of treatment. Abbreviations: 25Q—25th quartile; Me—median; 75Q—75th quartile; *—ANOVA Kruskal–Wallis test; **—Friedmann ANOVA test.

WHR [cm]	IPC Group	R-ESWT Group	F-ESWT Group	SC Group	p *	Post-Hoc
W1						IPC vs. SC
Me (25Q–75Q)	0.6 (0.4–1.4)	0.5 (0.3–1.1)	0.2 (0.0–0.8)	0.0 (0.0–0.3)	0.060	R-ESWT vs. SC
W2						
Me (25Q–75Q)	0.3 (−0.1–1.2)	0.7 (0.0–1.5)	0.2 (0.0–0.9)	0.3 (0.1–0.5)	0.767	-
W3						
Me (25Q–75Q)	0.6 (0.1–1.7)	0.3 (−0.38–1.1)	0.7 (−0.5–2.2)	0.3 (0.1–0.7)	0.174	-
W4						IPC vs. F-ESWT
Me (25Q–75Q)	1.0 (0.4–21.7)	0.5 (−0.3–0.8)	0.2 (0.5–0.6)	0.4 (0.2–0.5)	0.022	
*p* **	0.082	0.807	0.248	0.063		

## Data Availability

The data presented in this study are available on request from the corresponding author.
